# Effects of low-intensity pulsed ultrasound on muscle mass and *Fndc5* mRNA expression in aged male mice

**DOI:** 10.1007/s10522-025-10331-x

**Published:** 2025-10-03

**Authors:** Yoshitsugu Kojima

**Affiliations:** 1 Clinical Pharmacology Research Laboratory, Yokohama University of Pharmacy, 601 Matanocho Totsukaku, Yokohama, Kanagawa 245-0066 Japan; 2 Planning and Product Development Division, Nippon Sigmax Co., Ltd., 7Th Floor, 1-24-1 Nishi-Shinjuku, Shinjuku-ku, Tokyo, 160-0023 Japan

**Keywords:** Low-intensity pulsed ultrasound, Sarcopenia, *Fndc5*, Irisin, Muscle mass

## Abstract

Sarcopenia, the progressive loss of skeletal muscle mass and function with aging, is a growing public health concern. Conventional treatments such as exercise, pharmacological agents, and nutritional support offer limited efficacy, especially in older populations with reduced mobility or comorbidities. This study aimed to evaluate low-intensity pulsed ultrasound (LIPUS) as a novel, non-invasive therapeutic approach for age-related muscle atrophy. LIPUS was applied to the right hindlimbs of young (12-week), middle-aged (60-week), and aged (95-week) mice for 8 weeks. Muscle weights and mRNA expression levels were analyzed. In aged mice, LIPUS significantly increased gastrocnemius muscle mass and upregulated *Fndc5* and *Opa1* mRNA, genes associated with mitochondrial function and muscle regeneration. These findings suggest that LIPUS may serve as a safe, non-invasive intervention to counteract sarcopenia by promoting muscle growth and mitochondrial gene activation in aged skeletal muscle.

**Supplementary Information:**

The online version contains supplementary material available at 10.1007/s10522-025-10331-x.

## Introduction

Sarcopenia, an age-related decline in skeletal muscle mass and function, is a major contributor to frailty, falls, and loss of independence in older adults (Cruz-Jentoft et al. 2019). Despite its growing clinical relevance, effective interventions remain limited, with current strategies relying primarily on resistance training and nutritional supplementation. These approaches are often difficult to implement in frail or immobile individuals, highlighting the need for alternative, non-invasive therapies.

Recent studies have identified myokines—muscle-derived signaling molecules—as key mediators of muscle adaptation and systemic metabolic regulation. Among these, irisin, a cleavage product of the membrane protein FNDC5, has attracted attention for its role in promoting muscle hypertrophy and bone formation (Paoletti and Coccurello 2024; Colaianni et al. 2015). Importantly, irisin levels are modulated by physical activity and may be reduced in aging-related muscle decline.

Low-intensity pulsed ultrasound (LIPUS) is a non-invasive modality widely used to promote bone healing and tissue regeneration (Pilla et al. 1990; Bashardoust Tajali et al. 2012). Beyond its osteogenic effects, LIPUS has shown promise in enhancing muscle regeneration and preventing atrophy in various animal models (Tang et al. 2017; Jia et al. 2023). However, its potential to counteract sarcopenia has not been fully explored.

Given these multifaceted biological effects, I hypothesized that LIPUS irradiation could serve as a promising intervention to enhance skeletal muscle mass and myokine expression in sarcopenia. In this study, I employed a LIPUS protocol using a 2.0 MHz frequency, which has previously been demonstrated to promote muscle regeneration following injury (Mohamad Yusoff et al. 2023). This specific condition has also shown efficacy in clinical studies by enhancing angiogenesis in ischemic lower limbs (Mohamad Yusoff et al. 2021; Kajikawa et al. 2025; Mohamad Yusoff et al. 2025), suggesting its potential applicability to aged skeletal muscle. Therefore, I applied LIPUS to the hindlimb of aged mice, followed by analysis of muscle mass and myokine mRNA expression to evaluate its therapeutic potential.

## Methods

### Animals

Twelve-week-old (young), 60-week-old (middle), and 95-week-old (aged) male C57BL/6 mice (n = 24) were purchased from the Jackson Laboratory Japan, Inc. (Kanagawa, Japan) and acclimatized for 1 week before experiments. In C57BL/6 mice, 12–24 weeks of age corresponds approximately to 20–30 years in humans, 40–56 weeks to 40–50 years, and 72–104 weeks to 60–70 years, based on age equivalency models (Flurkey et al. 2007). The mice were housed under standard laboratory conditions with a 12-h light/dark cycle at 25 °C. Further, they were provided *ad libitum* access to water and food (Labo MR Stock, Nosan Corp., Kanagawa, Japan) containing 18.8% crude protein. All mice were fed the same food regardless of their age.

### LIPUS irradiation

**Fig. 1:**
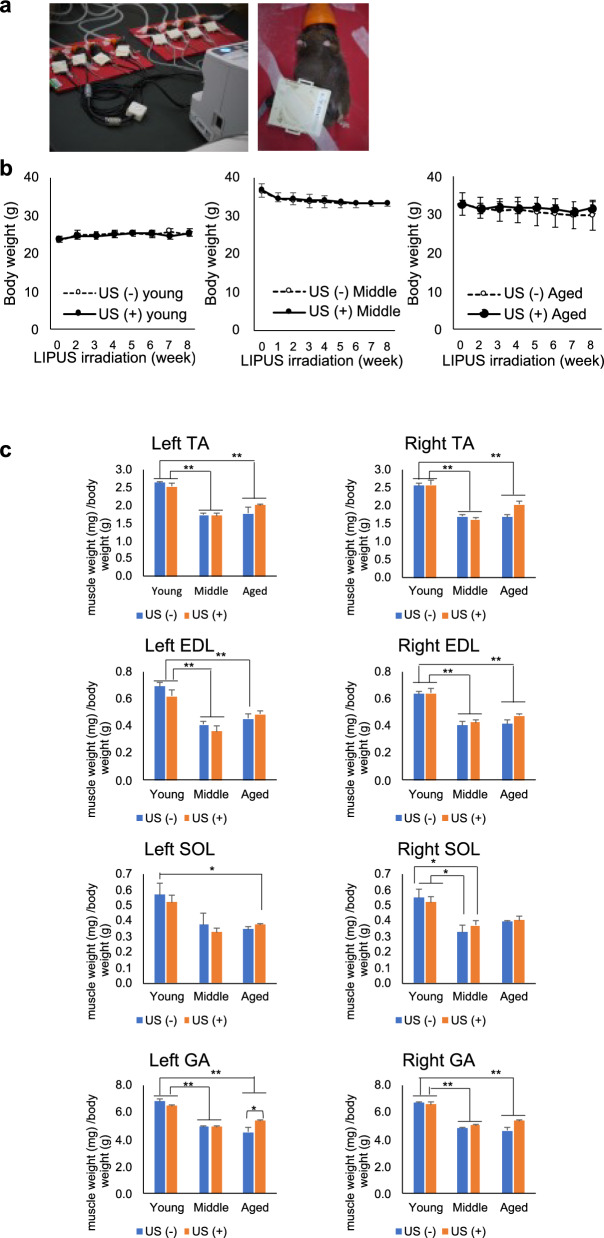

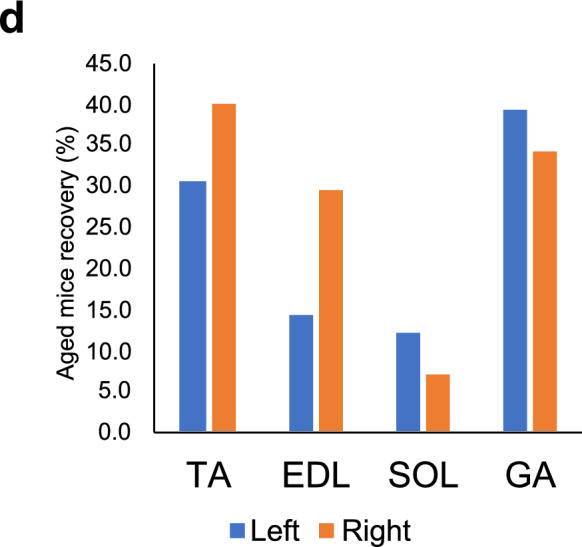
Low-intensity pulsed ultrasound (LIPUS) irradiation imaging and schematic study design. (**a**) Mice were anesthetized with isoflurane and placed on a heated pad at 35 °C. The transducer was placed over the skin of the right knee. LIPUS was administered for 20 min per day, 5 days per week for 8 weeks. (**b**) Graphs of the weight change in each group during LIPUS irradiation. Young: 12-week-old, Middle: 60-week-old, and Aged: 95-week-old male mice. (**c**) Wet weight of the lower limb muscles corrected for the body weight of each individual. Left = non-treated, Right = LIPUS irradiated. (**d**) Recovery rates were calculated relative to young controls to quantify the extent to which aged muscles approached youthful levels. Data are presented mean ± SEM. *p < 0.05; **p < 0.01 *vs.* non-US group for each muscle. Statistical analysis was performed using the paired t-test (**b**) and the two-way ANOVA followed by Tukey’s Honest Significant Difference post hoc test (**c**)

The young, middle, and aged mice were randomly assigned by body weight into the LIPUS (-) group (control) or LIPUS ( +) group (n = 4/group). A total of six groups were generated: LIPUS (-)/Young, LIPUS ( +)/Young, LIPUS (-)/Middle, LIPUS ( +)/Middle, LIPUS (-)/Aged, and LIPUS ( +)/Aged. Hair from the skin of the right hindlimb of each mouse was removed with depilatory cream (Reckitt Benckiser Japan Ltd., Tokyo, Japan) for LIPUS irradiation. The mice were anesthetized with isoflurane (Mylan Inc., Pittsburgh, PA, USA) using an animal anesthesia machine (DS Pharma Biomedical Co., Ltd., Osaka, Japan) (induction: concentration = 4%, flow rate = 2.8 L/min; maintenance: concentration = 2%, flow rate = 2.8 L/min). The anesthetized mice were placed on a heated pad maintained at 35 °C during the operation. Ultrasound gel was applied to an ultrasound transducer (Nippon Sigmax Co., Ltd., Tokyo, Japan), and the transducer was placed over the skin of the right knee (Fig. 1a). LIPUS treatment was administered 20 min per day, 5 days per week, for a total of 8 weeks. The ultrasound exposure conditions were as follows: effective transducer area, 6.85 cm^2^; intensity, 30 mW/cm^2^ with a 20% duty cycle; and pulse frequency, 2.0 MHz with a 1 kHz repeat rate. The mice in the LIPUS (-) group were anesthetized in the same manner as the mice in the LIPUS ( +) group, placed on a heat pad, and a transducer coated with ultrasound gel was placed on their right hindlimb. As a placebo treatment, no electricity was applied through the transducer. After 8 weeks of irradiation or sham treatment, blood samples were collected one day prior to euthanasia, and serum was harvested for subsequent analyses. The mice were euthanized by isoflurane overexposure to obtain lower limb skeletal muscles [tibialis anterior (TA), soleus (SOL), extensor digitorum longus (EDL), and gastrocnemius (GA)] for subsequent experiments.

### Immunohistochemical tissue staining

The right TA and right GA muscles were frozen in isopentane pre-chilled with liquid nitrogen and stored at -80 °C. After sectioning at 5-μm thickness, the muscle specimens were stained with anti-Laminin antibody (L9393, Sigma-Aldrich, St Louis, MO). Alexa Fluor 594 fluorescent dye conjugated to an anti-rabbit secondary antibody (A-11012, ThermoFisher Scientific, Waltham, MA) was used for visualization. Histopathological evaluations were performed with a fluorescence microscope (BZ-9000, Keyence, Osaka, Japan). The cross-sectional area (CSA) of muscle fibers was quantified using the Fiji imaging software (Schindelin et al. 2012). Individual muscle fibers were outlined manually traced using the polygon selection tool, and their areas were calculated with the 'Measure' function. For each sample, two to three representative fields were selected, and approximately 60 to 80 fibers were measured per field. In total, around 200 myofibers per sample were analyzed to assess CSA.

### Real-time polymerase chain reaction (PCR) analysis

The excised TA, SOL, EDL, and GA muscle samples were used for RNA extraction. After measuring wet muscle weight, the tissues were homogenized in TRIzol (ThermoFisher Scientific), and total RNA was isolated according to the manufacturer’s protocol (TRIzol). Briefly, chloroform was added into the TRIzol homogenized suspension. After centrifugation, the aqueous phase was collected in a new tube and added with an equal volume of isopropanol for precipitation. cDNA was synthesized using ReverTra Ace qPCR Kit (TOYOBO Co., Ltd., Osaka, Japan). The cDNAs were used as templates for real-time PCR reactions on a LightCycler 480 real-time PCR system (Roche Diagnostics Corp., Mannheim, Germany) using the THUNDERBIRD SYBR qPCR Mix (TOYOBO). The primer pairs are listed in Table 1.

**Table 1:** Primer sequences for real-time PCR

Gene	Forward Primer (5’-3’)	Reverse Primer (5’-3’)	Reference
*Interleukin-6*	atccagttgccttcttgggac	taagcctccgacttgtgaagt	Inda et al. (2010)
*Myostatin*	tggccatgatcttgctgtaa	ccttgacttctaaaaagggattca	Fu et al. (2018)
*Fndc5*^*a*^	atgaaggagatggggaggaa	gcggcagaagagagctataaca	Xiong et al. (2015)
*Pgc1*α^*b*^	agacaaatgtgcttcgaaaaagaa	gaagagataaagttgttggtttggc	Onder et al. (2019)
*Tfam*^*c*^	cacccagatgcaaaactttcag	ctgctctttatacttgctcacag	Sakakibara et al. (2016)
*Opa1*^*d*^	atactgggatctgctgttgg	aagtcaggcacaatccactt	Varanita et al. (2015)
*Gapdh*^*e*^	agcttgtcatcaacgggaag	tttgatgttagtggggtctcg	Acero-Bedoya et al. (2024)

^a^Fndc5: Fibronectin type III domain-containing protein 5

^b^Pgc1α: Peroxisome proliferator-activated receptor gamma coactivator 1-alpha

^c^Tfam: Transcription factor A, mitochondrial

^d^Opa1: Optic atrophy 1

^e^Gapdh: Glyceraldehyde-3-phosphate dehydrogenase

### Serum irisin enzyme-linked immunosorbent assay (ELISA)

Serum irisin concentrations were determined using a commercially available ELISA kit (Irisin, recombinant [Human, Rat, Mouse, Canine] – ELISA Kit; Phoenix Pharmaceuticals, Inc., Burlingame, CA, USA) according to the manufacturer’s protocol. Absorbance was measured at 450 nm using a microplate reader (DTX-880 Multimode Detector, Beckman Coulter, Brea, CA, USA).

### Micro-computed tomography (micro-CT)

At the end of the experiment, the right femora were harvested from the mice for micro-CT analysis (LCT-100Lite, Hitachi Aloka Medical, Ltd., Tokyo, Japan). Distal femora were measured until 7 mm from the distal end. The CT scans were performed with a tube voltage at 50 kV and tube current at 1 mA. The images were analyzed using Latheta software (Hitachi Aloka Medical) to outline the trabecular and cortical bone regions for each tomography slice.

### Statistical analysis

Parametric data are shown as mean ± standard error of the mean (SEM). *P*-values of < 0.05 were considered statistically significant. Two-way ANOVA was performed with post-hoc comparisons conducted using Tukey’s Honest Significant Difference (HSD) test. All statistical analyses were performed with EZR (Jichi Medical University, Tochigi, Japan), which is a graphical user interface for R (The R Foundation for Statistical Computing, Vienna, Austria). More precisely, it is a modified version of R commander designed to add statistical functions frequently used in biostatistics (Kanda 2013).

## Results

### LIPUS irradiation

Young mice (12-week old), middle-aged mice (60-week old), and aged mice (95-week old) were administered LIPUS on the right hindlimb for 8 weeks (Fig. 1a). During the LIPUS irradiation period, there were no significant differences in body weight between irradiated and non-irradiated mice (Fig. 1b). After 8 weeks of irradiation, the mice were euthanized to obtain both left (non-irradiated) and right (irradiated) hindlimb skeletal muscles, specifically the TA, SOL, EDL, and GA muscles, for muscle mass measurement and mRNA extraction. Two-way ANOVA revealed significant effects of both age and LIPUS intervention on muscle measurements in Right TA and Left GA. Post-hoc analysis using Tukey's test indicated a trend toward significance between the Aged US ( +) and Aged US ( −) groups in Right TA (*p* = 0.054), although the difference did not reach statistical significance. In contrast, a significant difference was observed between the Aged US ( +) and Aged US ( −) groups in Left GA (*p* = 0.025) (Fig. 1c). To quantify the extent to which LIPUS treatment restored aged muscle characteristics toward the young phenotype, a recovery rate (%) was calculated using the following formula (Fig. 1d):

Recovery (%) = (US [ +] Aged − US [ −] Aged) / (US [ −] Young − US [ −] Aged) × 100.

Based on this calculation, SOL muscles showed ~ 15% recovery bilaterally, while TA and GA muscles demonstrated > 30% recovery on both sides.

**Fig. 2:**
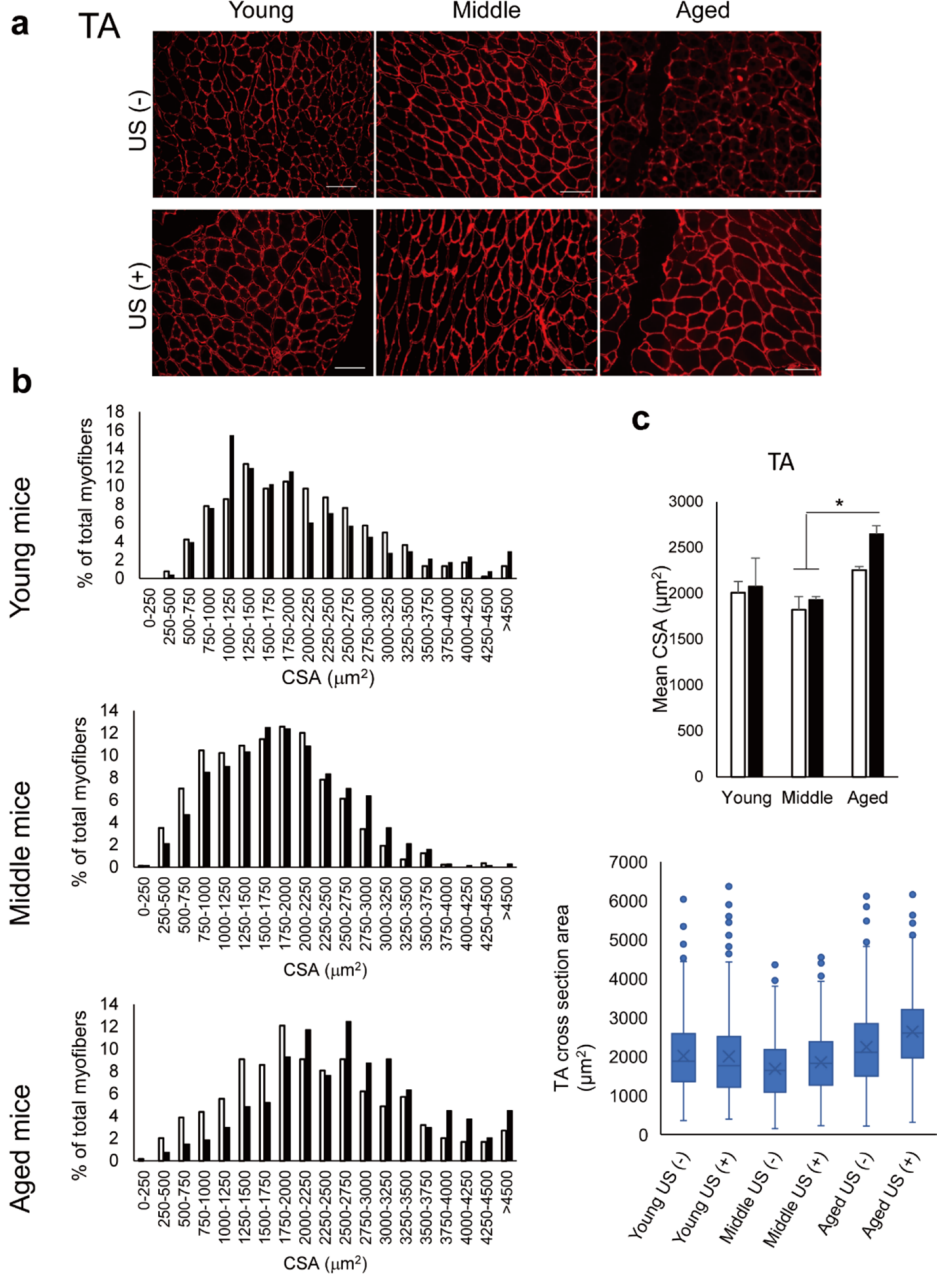

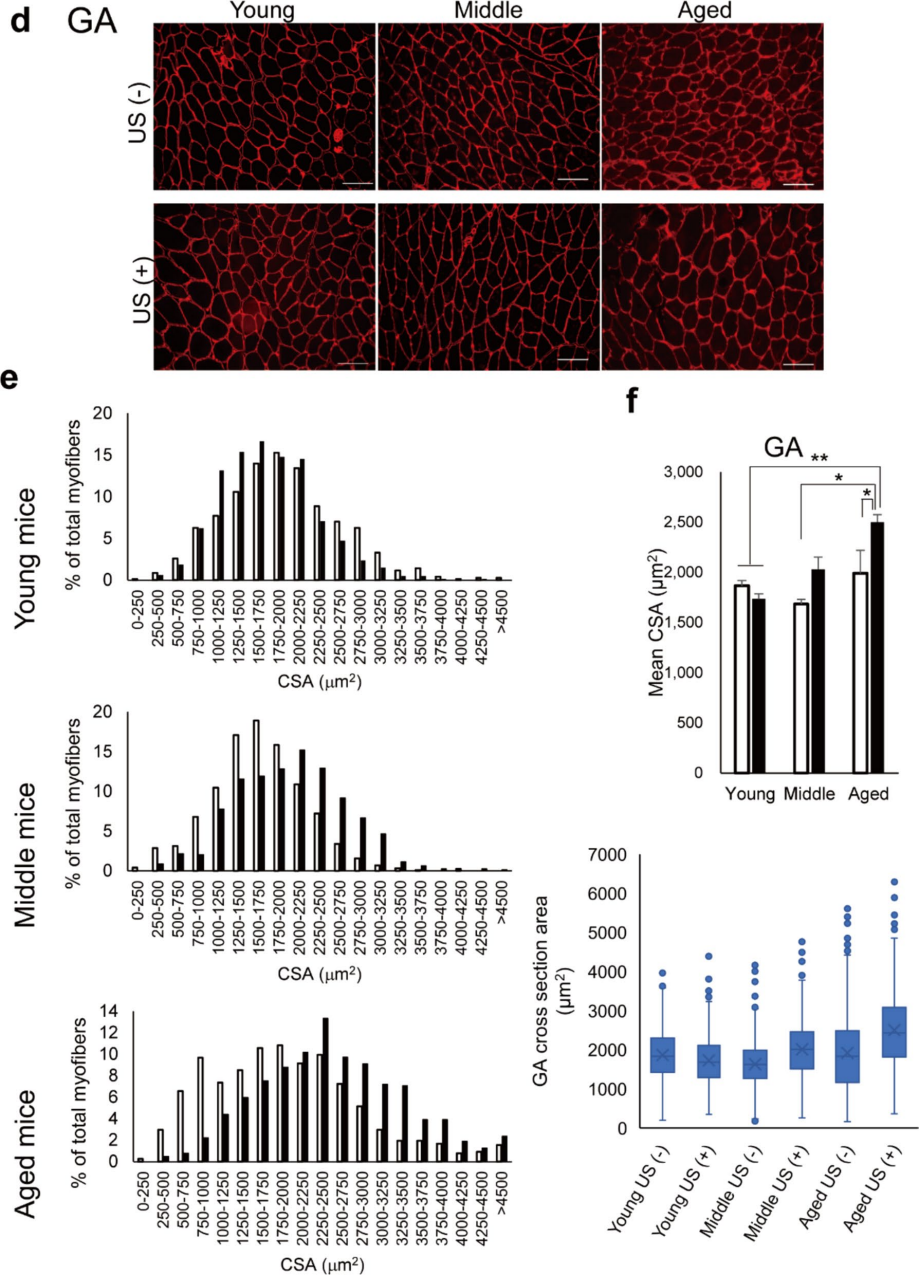
Effects of low-intensity pulsed ultrasound (LIPUS) on muscle fiber cross-sectional area (CSA) in the tibialis anterior (TA) and gastrocnemius (GA) muscles. Young: 12-week-old, Middle: 60-week-old, and Aged: 95-week-old male mice. (**a**) Representative histological images of laminin staining in the right TA, with (US +) or without LIPUS (US -). (**b**) Histogram of fiber CSA distribution of TA in the non-LIPUS group (white bar) and the LIPUS group (black bar). (**c**) The mean CSA of TA in the non-LIPUS group (white bar) and the LIPUS group (black bar). Box plots showing the CSA distribution of individual TA muscle fibers at the 25th, 50th, and 75th percentiles. (**d**) Representative histological images of laminin staining in right GA. (**e**) Histogram of fiber CSA distribution of GA in the non-LIPUS group (white bar) and the LIPUS group (black bar). (**f**) The mean CSA of GA in the non-LIPUS group (white bar) and the LIPUS group (black bar). Box plots showing the CSA distribution of individual GA muscle fibers at the 25th, 50th, and 75th percentiles. Data are presented mean ± SEM. *p < 0.05; **p < 0.01. For (**a**, **d**) scale bars = 100 μm. Statistical analysis was performed using the two-way ANOVA followed by Tukey’s Honest Significant Difference post hoc test (**c**, **f**)

To further investigate the impact of LIPUS on mice in each age group, the CSA of the TA (Fig. 2a-c) and GA (Fig. 2d-f) muscles was evaluated. The distribution of TA muscle fiber CSA was analyzed across six experimental groups: Young US (–), Young US ( +), Middle US (–), Middle US ( +), Aged US (–), and Aged US ( +). Box plot analysis revealed that the median CSA values tended to increase with age and were further elevated following ultrasound (US) treatment, particularly in the aged group. The interquartile range also expanded in aged muscles, indicating greater variability in fiber size. Notably, Aged US ( +) mice exhibited the highest median and upper quartile CSA values, suggesting a shift toward larger fiber populations in response to LIPUS stimulation. In the TA muscle, a two-way ANOVA revealed a significant main effect of age on mean CSA (F(2, 18) = 7.493, *p* = 0.0043). However, neither the main effect of LIPUS (F (1, 18) = 2.325, *p* = 0.145) nor the interaction between age and LIPUS (F(2, 18) = 0.665, *p* = 0.527) was statistically significant (Fig. 2c). In contrast, the GA muscle exhibited a different pattern. Two-way ANOVA revealed significant main effects of age (F(2, 18) = 8.915, *p* = 0.0020) and LIPUS (F(1, 18) = 6.548, *p* = 0.0197), as well as a significant interaction between the two factors (F(2, 18) = 4.193, *p* = 0.0320). A significant increase in mean CSA was observed in the LIPUS-treated group compared to the non-treated group, particularly in aged mice (Fig. 2f). To assess the distribution of muscle fiber CSA, the 25th, 50th (median), and 75th percentiles were calculated for each group. In the GA muscle, the 25th percentile values were 1422.53 µm^2^ in Young US (–), 1290.96 µm^2^ in Young US ( +), 1271.16 µm^2^ in Middle US (–), 1514.76 µm^2^ in Middle US ( +), 1165.37 µm^2^ in Aged US (–), and 1819.66 µm^2^ in Aged US ( +). Median CSA values (50th percentile) ranged from 1624.89 µm^2^ in Middle US (–) to 2434.90 µm^2^ in Aged US ( +), indicating a shift toward larger fiber sizes following LIPUS treatment in aged muscles. Similarly, the 75th percentile values increased from 1987.85 µm^2^ in Middle US (–) to 3083.77 µm^2^ in Aged US ( +), suggesting that LIPUS treatment promoted recovery of larger fiber populations in aged muscle. These findings indicate that both age and LIPUS independently and interactively influenced GA muscle CSA. Regarding the percent of myofibers, no difference was observed in young mice depending on the presence or absence of LIPUS irradiation. However, in the GA of middle-aged mice, larger fibers were observed in the LIPUS group compared to the non-LIPUS group. Furthermore, in aged mice, larger fibers were observed in the LIPUS group compared to the non-LIPUS group in both TA and GA. In aged mice, muscle fibers appeared morphologically heterogeneous compared to those in young and middle-aged mice. Notably, LIPUS-treated aged mice exhibited more uniform fiber shapes, suggesting a potential structural stabilizing effect of LIPUS on aged skeletal muscle.

### Real-time PCR analysis

**Fig. 3:**
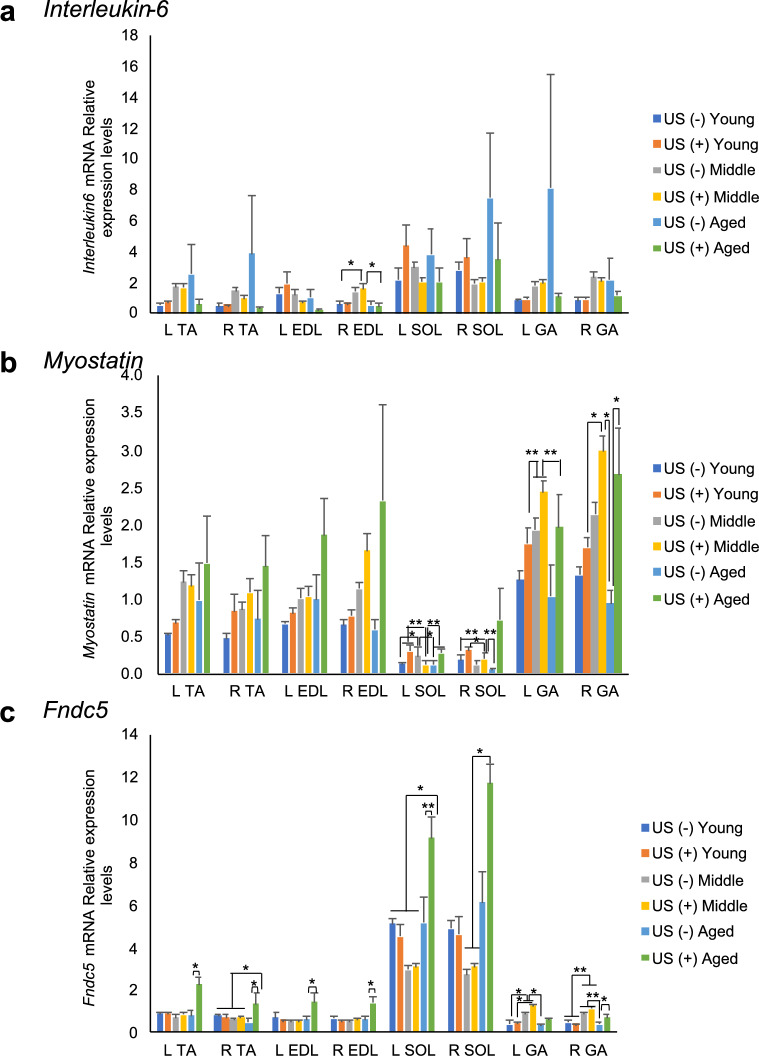

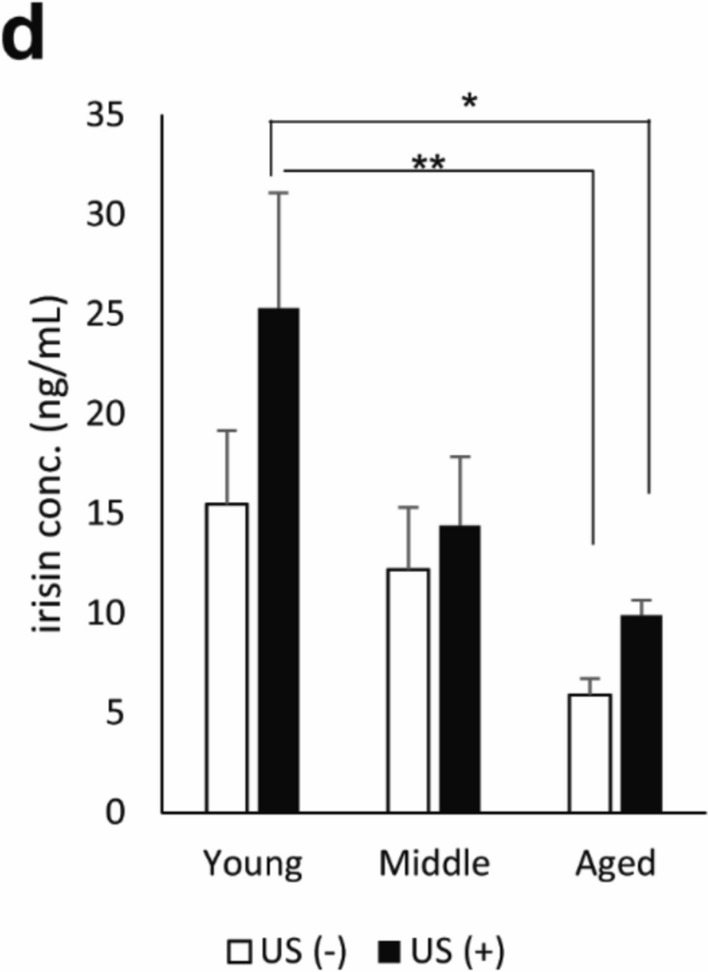
Changes in the mRNA levels of myokines with (US +) or without LIPUS treatment (US-) (n = 4/group). Young: 12-week-old, Middle: 60-week-old, and Aged: 95-week-old male mice. (**a**) *Interleukin-6*, (**b**) *Myostatin*, (**c**) *Fndc5*. (**d**) Serum irisin levels measured by ELISA following LIPUS treatment. Data are presented mean ± SEM. *p < 0.05; **p < 0.01. Statistical analysis was performed using the two-way ANOVA followed by Tukey’s Honest Significant Difference post hoc test

To investigate the effect of LIPUS irradiation on myokines, we analyzed the mRNA expression levels of myokines, *IL-6*, *Myostatin*, and *Fndc5*, by real-time PCR (Fig. 3a-c). The *IL-6* expression levels were not affected by LIPUS irradiation in all age groups. Surprisingly, *Fndc5* mRNA levels were significantly increased in almost all muscles of aged mice with LIPUS treatment (Fig. 3c). FNDC5 undergoes cleavage to generate irisin, a myokine that is secreted into the bloodstream (Maak et al. 2021). To further assess the effects of LIPUS treatment, serum irisin concentrations were measured across age groups (Fig. 3d). Two-way ANOVA revealed a significant main effect of age, with irisin levels decreasing with aging (*p* = 0.006). Post hoc analysis using Tukey’s HSD indicated that young mice in the LIPUS-treated group (US [ +]) exhibited significantly higher irisin concentrations compared to aged mice in both the US ( +) and US ( −) groups (vs. US [ +] aged: *p* = 0.0489; vs. US [ −] aged: *p* = 0.0087).

However, neither the main effect of LIPUS treatment nor its interaction with age reached statistical significance, suggesting that the independent contribution of LIPUS to irisin elevation remains inconclusive. Notably, although two-way ANOVA did not detect significant treatment effects, student t-tests revealed differences between LIPUS and control groups within specific age categories (see Supplementary Figure S2), warranting further investigation.

Irisin affects bone and adipose tissue (Maak et al. 2021). Therefore, to determine if increased mRNA expression of *Fndc5* impacted bone morphology, bone density of isolated femurs was measured using micro-CT. However, there was no significant differences in bone density between the groups with and without LIPUS irradiation (Supplemental Tables 1 and 2).

**Fig. 4:**
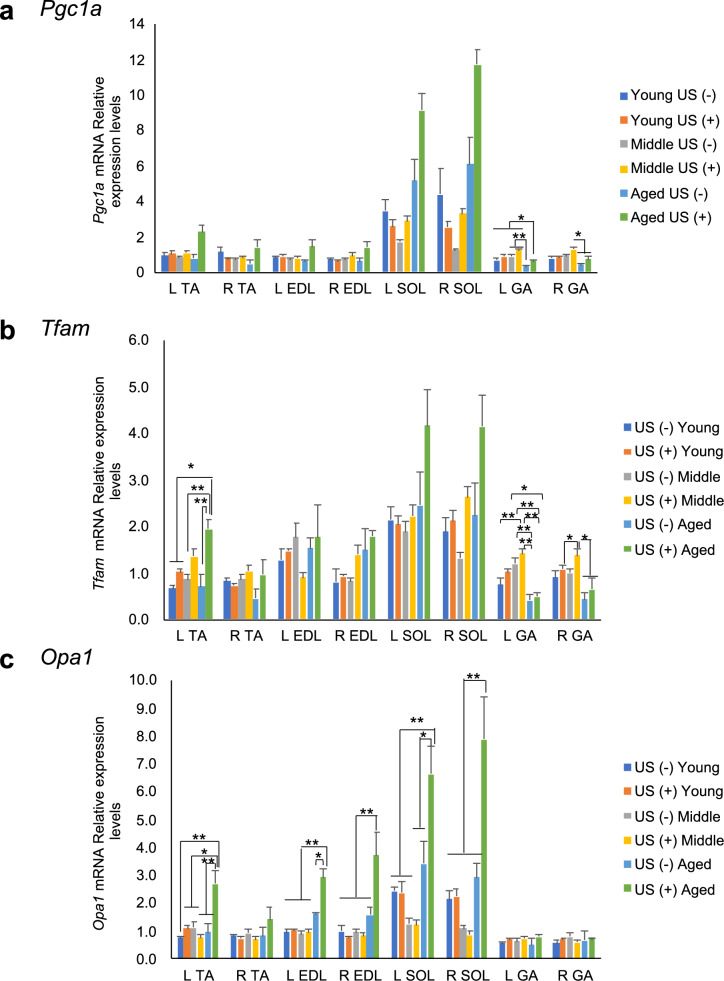
Changes in the mRNA levels of *Pgc1a* and mitochondrial metabolism-related factors with (US +) or without LIPUS treatment (US-) (n = 4/group). (**a**) *Pgc1a*, (**b**) *Tfam*, and (**c**) *Opa1*. Young: 12-week-old, Middle: 60-week-old, and Aged: 95-week-old male mice. Data are presented as mean ± SEM. *p < 0.05; **p < 0.01. Statistical analysis was performed using the two-way ANOVA followed by Tukey’s Honest Significant Difference (HSD) post hoc test

We further analyzed the mRNA expression levels of *Pgc1α*, one of the key regulators of *Fndc5* gene expression. Two-way ANOVA revealed no significant difference in Pgc1α mRNA expression following LIPUS treatment in any age group (Fig. 4a). Next, I analyzed the expression of *Tfam*, whose expression is regulated by PGC-1α. *Tfam* mRNA expression was significantly increased in the TA of aged mice following LIPUS treatment (Fig. 4b). Furthermore, analysis of the mRNA expression levels of OPA1, which controls mitochondrial fusion, revealed no significant difference in young- and middle-aged mice with or without LIPUS irradiation treatment (Fig. 4c). In contrast, aged mice showed a significant upregulation of *Opa1* mRNA in multiple muscles following LIPUS treatment, including the left TA, left and right EDL, and left and right SOL. These results suggest that LIPUS irradiation selectively activates mitochondrial regulatory pathways in aged skeletal muscle, primarily through the upregulation of PGC-1α downstream targets such as *Opa1*. The absence of significant changes in young and middle-aged mice highlights an age-dependent responsiveness, indicating that aged muscle may be more susceptible to LIPUS-induced metabolic modulation.

## Discussion

This study provides compelling evidence that LIPUS may serve as a promising non-invasive intervention for sarcopenia. In aged mice, LIPUS irradiation significantly increased muscle mass, particularly in fast-twitch fiber-rich muscles such as the left GA. Although the right TA did not reach statistical significance, a similar trend was observed. These muscles play a critical role in toe clearance and fall prevention (Perera et al. 2021; Kim et al. 2022), underscoring the functional relevance of the observed hypertrophy.

Mechanistically, LIPUS treatment upregulated *Fndc5* mRNA expression across multiple muscle types in aged mice, suggesting that irisin—a cleavage product of FNDC5—may mediate the anabolic effects of LIPUS. Supporting this hypothesis, *Opa1* mRNA, which is regulated by irisin and involved in mitochondrial fusion, was significantly elevated in the left TA, bilateral EDL, and SOL muscles. These findings point to activation of the PGC-1α–FNDC5–irisin axis by LIPUS, contributing to muscle regeneration and metabolic enhancement in aged skeletal muscle.

Interestingly, despite the upregulation of *Fndc5*, *Pgc1α* mRNA itself did not show a statistically significant increase following LIPUS treatment, as determined by two-way ANOVA. This discrepancy may reflect post-transcriptional regulation or tissue-specific responsiveness. Notably, LIPUS was applied to the right limb, yet molecular changes were observed bilaterally, suggesting that irisin may exert paracrine effects via circulation.

In contrast, young mice did not exhibit significant changes in muscle mass or molecular markers such as *Pgc1α*, *Tfam*, or *Opa1*, consistent with previous human studies indicating that FNDC5 expression is more responsive to endurance stimuli in older individuals (Huh et al. 2012). This age-dependent responsiveness may reflect a broader therapeutic window for LIPUS in aging populations. Several factors may explain the lack of effect in young mice: high baseline muscle mass and activity levels, elevated expression of anabolic regulators, reduced inflammation, and naturally high satellite cell activity—all of which may limit the observable impact of LIPUS.

Additionally, aged mice showed increased CSA in certain muscle fibers compared to young mice, possibly reflecting a compensatory hypertrophic response to age-related fiber loss. This structural remodeling may help preserve muscle function despite reduced fiber number (Lee et al. 2022).

Although LIPUS has previously been shown to improve bone mineral density in ovariectomized mice (Kojima et al. 2024), no such effect was observed in this study. Differences in irradiation frequency may account for this discrepancy, as prior studies suggest that less frequent LIPUS exposure may be more effective for bone outcomes.

Beyond its anabolic effects, LIPUS offers several practical advantages. Its transcutaneous application enables safe, repeatable use, with potential for home-based therapy. LIPUS may also mitigate inflammaging by promoting macrophage polarization toward the anti-inflammatory M2 phenotype and suppressing pro-inflammatory cytokines (Chen et al. 2023). Furthermore, it has been reported to enhance local blood flow and vasodilation, improving oxygen and nutrient delivery to muscle tissues (Kajikawa et al. 2025; Mohamad Yusoff et al. 2025). Mechanical modulation of macrophage function may represent a novel strategy for combating age-related muscle degeneration.

However, several limitations must be acknowledged. The localized nature of LIPUS may require multi-transducer systems for broader application. Most current evidence is based on mRNA expression; further validation at the protein level—such as irisin quantification via ELISA—is needed. In this study, although a t-test showed significant differences in serum irisin levels, two-way ANOVA did not detect a significant main effect or interaction, leaving the independent contribution of LIPUS to irisin elevation inconclusive (see Supplementary Fig. S2).

Study design limitations also warrant consideration. Only male mice were used, and sex differences in sarcopenia should be explored in future research. The study included six experimental groups based on three age categories (young, middle-aged, aged) and two intervention conditions (LIPUS vs. control), with four animals per group (n = 4). A post hoc power analysis for two-way ANOVA, assuming a moderate effect size (Cohen’s f = 0.25) and α = 0.05, yielded a statistical power of approximately 10.6%, indicating limited sensitivity to detect group differences. Given the exploratory nature of this pilot study and ethical constraints on animal use, the sample size was minimized. These findings will inform the design of future studies with larger sample sizes and improved statistical power.

Since a single transducer can only affect a portion of the lower limb muscles with ultrasound, using a device equipped with eight transducers designed to encircle calf, which has already been developed for lower limb ischemia treatment, may potentially demonstrate effects more suitable for clinical application (Mohamad Yusoff et al. 2021; Kajikawa et al. 2025; Mohamad Yusoff et al. 2025).

In conclusion, our findings support LIPUS as a promising, non-invasive intervention for sarcopenia. By potentially activating the PGC-1α–FNDC5–irisin pathway, LIPUS may enhance muscle mass and mitochondrial function in aged skeletal muscle. These results lay the groundwork for future translational studies aimed at developing LIPUS-based therapies for age-related muscle degeneration.

## Supplementary Information

Below is the link to the electronic supplementary material.

Supplementary file1 (TIF 23423 KB)

Supplementary file2 Supplementary Fig. 2 Serum irisin levels measured by ELISA following LIPUS treatment. Data are presented mean ± SEM. *p<0.05 vs. non-US group for each muscle. Statistical analysis was performed using the Student’s t-test (TIF 10224 KB)

Supplementary file3 (DOCX 22 KB)

## Data Availability

No datasets were generated or analysed during the current study.

## Abbreviations

EDL: Extensor digitorum longus
GA: Gastrocnemius
IL-6: Interleukin-6
LIPUS: Low-intensity pulsed ultrasound
microCT: Micro-computed tomography
SEM: Standard error of the mean
SOL: Soleus
TA: Tibialis anterior
OVX: Ovariectomized
