# Supplementary figures and images for: Effects of low-intensity pulsed ultrasound on muscle mass and Fndc5 mRNA expression in aged male mice
Source: Biogerontology. 2025 Oct 3;26(5):187. doi: 10.1007/s10522-025-10331-x (PMC12494648; doi:10.1007/s10522-025-10331-x)

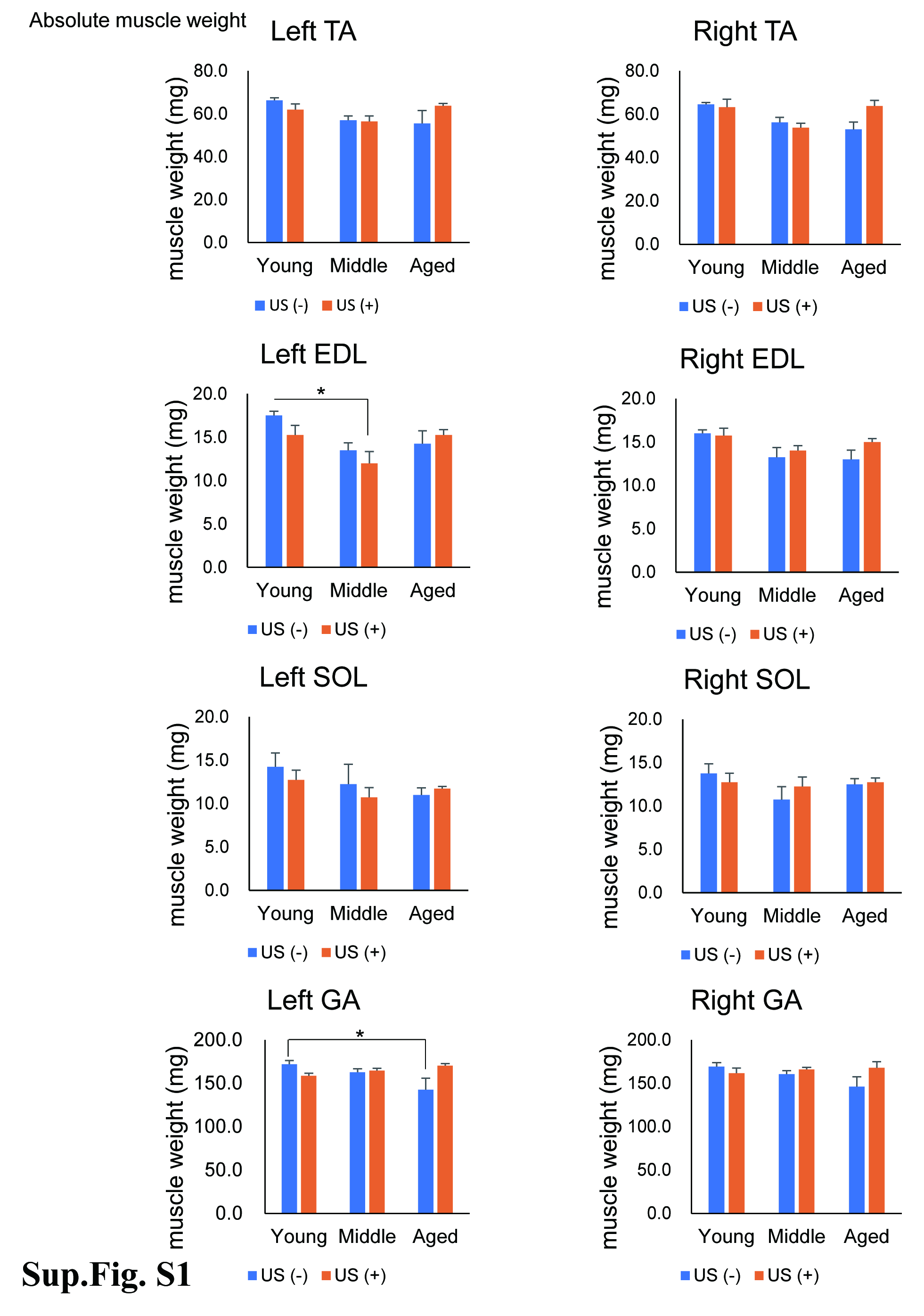

Supplement: Supplementary file 1 — Supplementary file1 (TIF 23423 KB) [file 10522_2025_10331_MOESM1_ESM.tif]

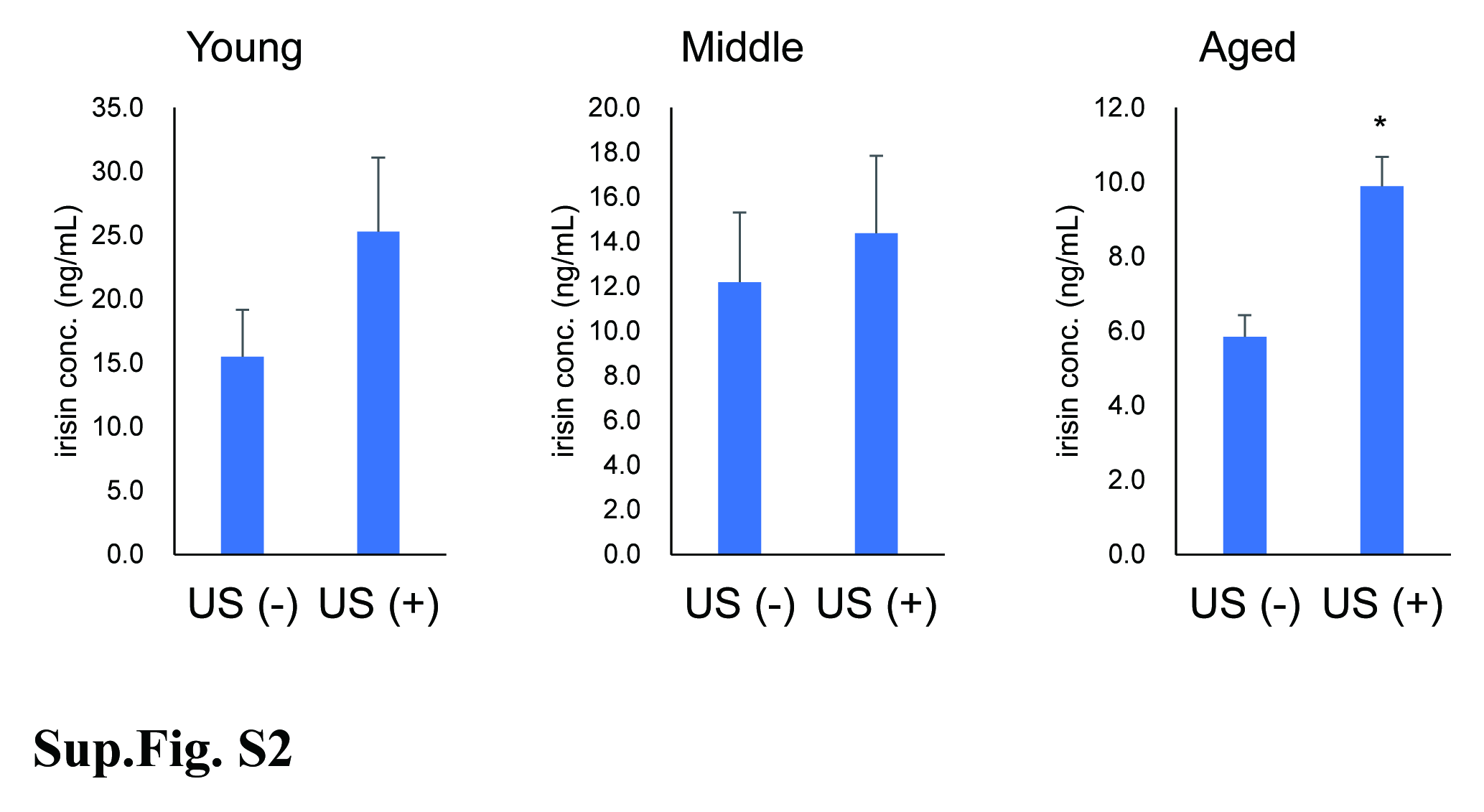

Supplement: Supplementary file 2 — Supplementary file2 Supplementary Fig. 2 Serum irisin levels measured by ELISA following LIPUS treatment. Data are presented mean ± SEM. *p<0.05 vs. non-US group for each muscle. Statistical analysis was performed using the Student’s t-test (TIF 10224 KB) [file 10522_2025_10331_MOESM2_ESM.tif]
