# Supplementary material for: Effects of low-intensity pulsed ultrasound on muscle mass and Fndc5 mRNA expression in aged male mice
Source: Biogerontology. 2025 Oct 3;26(5):187. doi: 10.1007/s10522-025-10331-x (PMC12494648; doi:10.1007/s10522-025-10331-x)
Supplement: Supplementary file 3 — Supplementary file3 (DOCX 22 KB) [file 10522_2025_10331_MOESM3_ESM.docx]

**Supplemental Information**

Effects of low-intensity pulsed ultrasound on muscle mass and Fndc5 mRNA expression in aged mice

**Yoshitsugu Kojima^1, 2*^**

^1^Clinical Pharmacology Research Laboratory, Yokohama University of Pharmacy, 601 Matanocho Totsukaku, Yokohama, Kanagawa, Japan, 245-0066

^2^Planning and Product Development Division, Nippon Sigmax Co., Ltd., Shinjuku-ku, Tokyo, Japan

Email: yoshi_kojima@sigmax.co.jp

**Supplemental Table 1** Cortical bone mineral density of the femora measured by microCT

|  | Young | |  | Middle | |  | Aged | |
| --- | --- | --- | --- | --- | --- | --- | --- | --- |
|  | Left | Right |  | Left | Right |  | Left | Right |
| LIPUS (-) | 886.8 ± 9.5 | 889.0 ± 7.1 |  | 897.9 ± 17.8 | 876.2 ± 25.3 |  | 776.2 ± 60.5 | 741. 1 ± 56.7 |
| LIPUS (+) | 880.7 ± 1.6 | 880.3 ± 4.8 |  | 930.3 ± 19.0 | 912.5 ± 22.8 |  | 741.9 ± 28.4 | 723.9 ± 38.3 |

Cortical bone mineral density (mg/cm^3^) data are presented as mean ± SEM (n = 4/group).

LIPUS = low-intensity pulsed ultrasound, Young = 12 week-old mice group, Middle = 60 week-old mice group, Aged = 95 week-old mice group. Left = non-treated, Right = LIPUS irradiated. In the LIPUS (-) group, irradiation was a placebo treatment.

**Supplemental Table 2** Trabecular bone mineral density of the femora measured by microCT

|  | Young | |  | Middle | |  | Aged | |
| --- | --- | --- | --- | --- | --- | --- | --- | --- |
|  | Left | Right |  | Left | Right |  | Left | Right |
| LIPUS (-) | 325.7 ± 8.4 | 321.4 ± 7.4 |  | 260.6 ± 16.4 | 275.7 ± 13.7 |  | 245.7 ± 19.3 | 226.8 ± 23.5 |
| LIPUS (+) | 314.4 ± 5.8 | 317.5 ± 2.7 |  | 276.0 ± 13.7 | 284.0 ± 8.9 |  | 199.3 ± 16.0 | 222.0 ± 12.9 |

Trabecular bone mineral density (mg/cm^3^) values are presented as mean ± SEM (n = 4/ group). LIPUS = low-intensity pulsed ultrasound, Young = 12 week-old mice group, Middle = 60 week-old mice group, Aged = 95 week-old mice group. Left = non-treated, Right = LIPUS irradiated. In the LIPUS (-) group, irradiation was a placebo treatment.
